# Radiologic Characterization of Invasive Fungal Infections of the Paranasal Sinuses and Skull Base: A Prospective Analysis

**DOI:** 10.7759/cureus.104104

**Published:** 2026-02-23

**Authors:** Shamsuddoha S, Surya Kant, Satyavrat Verma, Anurag Singh, Vivek Singh, Amit Keshri, Anil Singh, Rungmei S K Marak, Sheo Kumar

**Affiliations:** 1 Radiodiagnosis, Sanjay Gandhi Postgraduate Institute of Medical Sciences, Lucknow, IND; 2 Pathology, Sanjay Gandhi Postgraduate Institute of Medical Sciences, Lucknow, IND; 3 Head and Neck Surgery, Sanjay Gandhi Postgraduate Institute of Medical Sciences, Lucknow, IND; 4 Microbiology, Sanjay Gandhi Postgraduate Institute of Medical Sciences, Lucknow, IND

**Keywords:** aspergillosis, invasive fungal sinusitis, mucormycosis, sinus, skull base

## Abstract

Background: Invasive sinonasal and skull base fungal infections represent life-threatening conditions with increasing incidence. Early diagnosis and correct delineation of disease extent using imaging modalities are crucial for reducing morbidity and mortality.

Aim and objectives: The aim and objectives of the study are to analyze and compare the computed tomography and magnetic resonance imaging features of acute and chronic invasive sinonasal and skull base fungal infections, correlate imaging findings with microbiological and histopathological findings, and evaluate post-treatment imaging changes on follow-up radiological findings.

Materials and methods: This prospective study included 60 patients with proven fungal infections (30 acute invasive and 30 chronic invasive fungal infections). All patients underwent radiological examination of the paranasal sinuses, skull base, orbit, and brain. Imaging findings were meticulously noted, correlated with tissue diagnosis, and compared between both acute and chronic groups. Follow-up imaging was performed at three months (for acute infections) and six months (for chronic infections).

Results: Acute invasive fungal infections predominantly affected immunocompromised patients (76.7% (23/30)), while chronic invasive infections were more common in immunocompetent individuals (80% (24/30)). The maxillary sinus was the most frequently involved sinus in both groups. Orbital, skull base, cavernous sinus, and intracranial involvement were significantly more common in acute invasive infections (p < 0.05). Follow-up imaging demonstrated radiological improvement in 76.7% (23/30) of acute and 83.3% (25/30) of chronic cases, with no statistically significant difference between the two groups.

Conclusion: Computed tomography and magnetic resonance imaging play vital and indispensable roles in the diagnosis, staging, and follow-up of invasive sinonasal and skull base fungal infections. Recognition of characteristic imaging patterns makes early diagnosis, guides surgical and medical management, and helps in determining the duration of antifungal therapy.

## Introduction

Invasive fungal infections involving the sinonasal cavities and skull base were not common in the past but have shown a marked rise in incidence over the last decade, particularly in developing countries such as India. The surge has been attributed to the increasing prevalence of diabetes mellitus, the widespread use of corticosteroids and immunosuppressive agents, organ transplantation, malignancy-related immunosuppression, and, more recently, coronavirus disease 2019 (COVID-19)-associated immune dysregulation [1-3]. These infections are associated with aggressive local invasion, angioinvasion, tissue necrosis, and a high risk of orbital and intracranial complications, resulting in significant morbidity and mortality.

According to the DeShazo classification, fungal rhinosinusitis is categorized into invasive and non-invasive forms. Invasive fungal rhinosinusitis includes acute invasive, chronic invasive, and chronic granulomatous invasive disease, while non-invasive forms include allergic fungal rhinosinusitis and fungal ball [4]. Invasive disease is defined by histopathological evidence of fungal hyphae invading mucosa, submucosa, bone, or blood vessels.

Imaging plays a pivotal role in the early diagnosis of invasive fungal infections, often preceding overt clinical deterioration. Computed tomography (CT) is particularly useful for detecting bony erosions and hyperdense fungal elements, whereas magnetic resonance imaging (MRI) provides superior evaluation of soft tissue extension, vascular invasion, orbital involvement, and intracranial complications [5-7]. Despite numerous studies describing imaging findings in acute invasive fungal sinusitis, comparative data evaluating imaging patterns and treatment response between acute and chronic invasive disease remain limited. The present study aims to address this gap.

The objectives of this study were to diagnose invasive sinonasal and skull base fungal infections using CT and/or MRI with definitive diagnosis of histopathology and/or microbiological confirmation, to describe and compare the imaging features of acute and chronic invasive fungal infections, to assess post-treatment radiological changes on follow-up imaging, and to aid in determining the appropriate duration of antifungal therapy and imaging surveillance.

## Materials and methods

Study design and population

**Figure 1:**
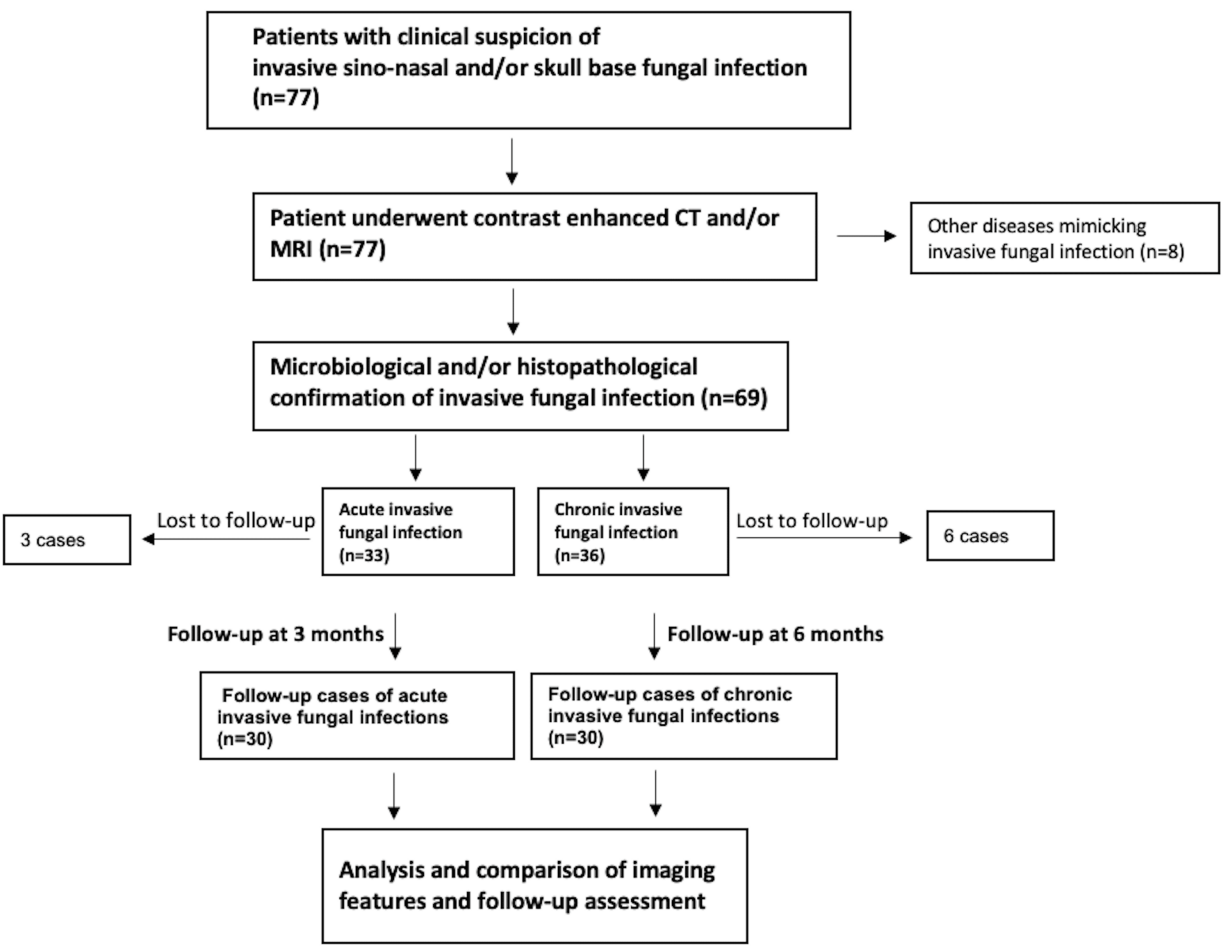
Illustrative flowchart to demonstrate the patient selection process. CT: computed tomography; MRI: magnetic resonance imaging

The present prospective study was conducted at the Department of Radiodiagnosis, Sanjay Gandhi Postgraduate Institute of Medical Sciences (SGPGIMS), Lucknow, in collaboration with the Departments of Neurosurgery, Pathology, and Microbiology over the period of two years from November 2019 to October 2021. Sixty cases with microbiologically and/or histopathologically proven invasive sinonasal and skull base fungal infections were included after obtaining informed consent and the approval of the institutional ethics committee (Figure 1).

Inclusion and exclusion criteria

A total of 77 patients with clinical suspicion of invasive sinonasal and/or skull base fungal infection underwent CT and/or MRI. Based on imaging findings with microbiological and/or histopathological confirmation, 36 cases were diagnosed with chronic invasive fungal infection and 33 cases with acute invasive fungal infection, and eight cases were found to have other diseases mimicking invasive sinonasal and/or skull base fungal infection (sinonasal squamous cell carcinoma, sinonasal lymphoma, and bacterial skull base osteomyelitis) and were excluded from further analysis. During the follow-up, six patients from the chronic invasive group and three patients from the acute invasive group were lost to follow-up and were also excluded from the study. Consequently, 30 patients with acute invasive fungal infection, who completed the three-month follow-up, and 30 patients with chronic invasive fungal infection, who completed the six-month follow-up, were finally included in the study.

Acute invasive fungal infection was characterized by a rapidly progressive fungal infection of the sinonasal cavities and/or skull base with a duration of symptoms ≤ 4 weeks and showed histological evidence of fungal hyphae with aggressive clinical and imaging features. Chronic invasive fungal infection was characterized as a slowly progressive fungal infection with a symptom duration of >4 weeks, showing histopathological presence of fungal hyphae with chronic inflammatory response and relatively indolent clinical and imaging features compared to acute invasive fungal infection.

Imaging protocol

CT examinations were performed using a 64-slice multidetector CT scan (Brilliance 190 P 64-channel CT scanner, Philips, Amsterdam, The Netherlands) or a 128-slice multidetector CT scan (SOMATOM Definition AS+ 64544, Siemens, Malvern, PA, USA) with acquisition in axial planes and multiplanar reconstructions. MRI was performed on a 3 T MR scanner (Signa HDXT, General Electric Healthcare Technologies, Milwaukee, WI, USA) using standard head and paranasal sinus protocols, including T1-weighted, T2-weighted, fat-suppressed, diffusion-weighted, susceptibility-weighted, and post-contrast sequences.

**Figure 2:**
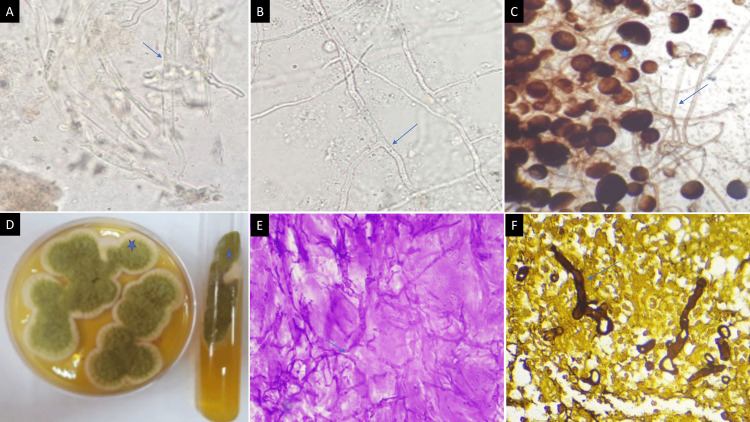
(A) 10% KOH wet mount of sinonasal biopsy tissue debris displaying plenty of broad aseptate branching fungal hyphae of mucormycetes (↗) (40x). (B) Plenty of thin hyaline septate acute-angled branching fungal hyphae of Aspergillus (↗) (40x). (C) Lactophenol cotton blue mount of mucormycetes culture shows branched sporangiophores terminating in sporangium with subspherical columella containing brownish sporangiospores (*) with primitive rhizoids of Rhizomucor pusillus (↗). (D) Culture on Sabouraud's dextrose agar plate and tube (37°C) shows velvety powdery greenish-yellow growth of Aspergillus flavus (*). (E) Periodic acid-Schiff-stained histopathology section displaying thin hyaline septate acute-angled branching fungal hyphae of Aspergillus (↗) (40x). (F) Silver methenamine-stained histopathology section displaying broad aseptate branching fungal hyphae of mucormycetes (↗) (40x).

Microbiological and/or histopathological confirmation was obtained in all cases. Direct microscopy using a 10% KOH mount, lactophenol cotton blue mount, and fungal culture on Sabouraud’s dextrose agar identified *Mucorales* and *Aspergillus* species. Broad aseptate hyphae with right-angled branching were seen in mucormycosis, while thin septate acute-angled branching hyphae were characteristic of aspergillosis (Figures 2A-2D). Histopathological examination of biopsy specimens showed diffuse tissue necrosis with invasion by fungal elements (Figures 2E, 2F). These findings confirmed fungal infection and correlated with imaging features of the disease.

Follow-up assessment

Acute fungal infections are rapidly progressive and aggressive in nature and potentially life-threatening; therefore, a short three-month follow-up was used to assess early response and detect disease status. Chronic fungal infections, on the other hand, are indolent, slow-growing processes and take a longer time to show up radiologically; thus, a longer follow-up at six months was done to better evaluate therapy response. Radiological response was assessed based on reduction in the size or number of lesions, normalization of signal or attenuation findings, reduction in enhancement, stabilization or healing of bony erosions, and correlation with clinical improvement.

Statistical analysis

Statistical analysis was performed using IBM SPSS Statistics for Windows, version 25.0 (IBM Corp., Armonk, NY, USA). Continuous variables were expressed as mean ± standard deviation and compared using independent sample t-tests. Categorical variables were analyzed using chi-squared or Fisher’s exact tests. A p-value < 0.05 was considered statistically significant.

## Results

Demographic and clinical characteristics

The mean age of patients with acute invasive fungal infection was significantly higher than that of patients with chronic invasive infection (50.17 ± 11.2 years vs. 41.53 ± 14.27 years; p < 0.01). Acute invasive disease showed a male predominance, whereas chronic invasive disease displayed a relatively balanced gender predisposition. Immunological status differed significantly between the two groups: 76.7% (23/30) of acute invasive cases were immunocompromised, while 80% (24/30) of chronic invasive cases were immunocompetent, displaying differing host susceptibility patterns (Table 1).

**Table 1:** Demographic and immunological profile of the study population.

Parameter	Acute invasive fungal infection (n = 30)	Chronic invasive fungal infection (n = 30)	p-value
Mean age (years)	50.17 ± 11.2	41.53 ± 14.27	<0.01
Male	20 (66.6%)	16 (53.3%)	0.612
Female	10 (33.3%)	14 (46.7%)	0.612
Immunocompromised	23 (76.7%)	6 (20%)	<0.001
Immunocompetent	7 (23.3%)	24 (80%)	<0.001

Distribution of anatomical sites involved

**Figure 3:**
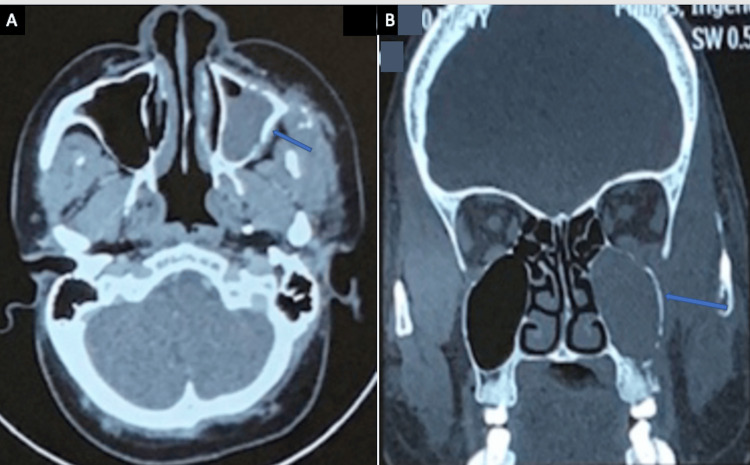
(A) A case of acute invasive sinonasal fungal infection showing left maxillary sinus infiltration (↗) (CT scan, axial). (B) CT scan coronal bone window highlighting left posterior maxillary wall erosion (↗) (CT scan, coronal bone window). CT: computed tomography

**Figure 4:**
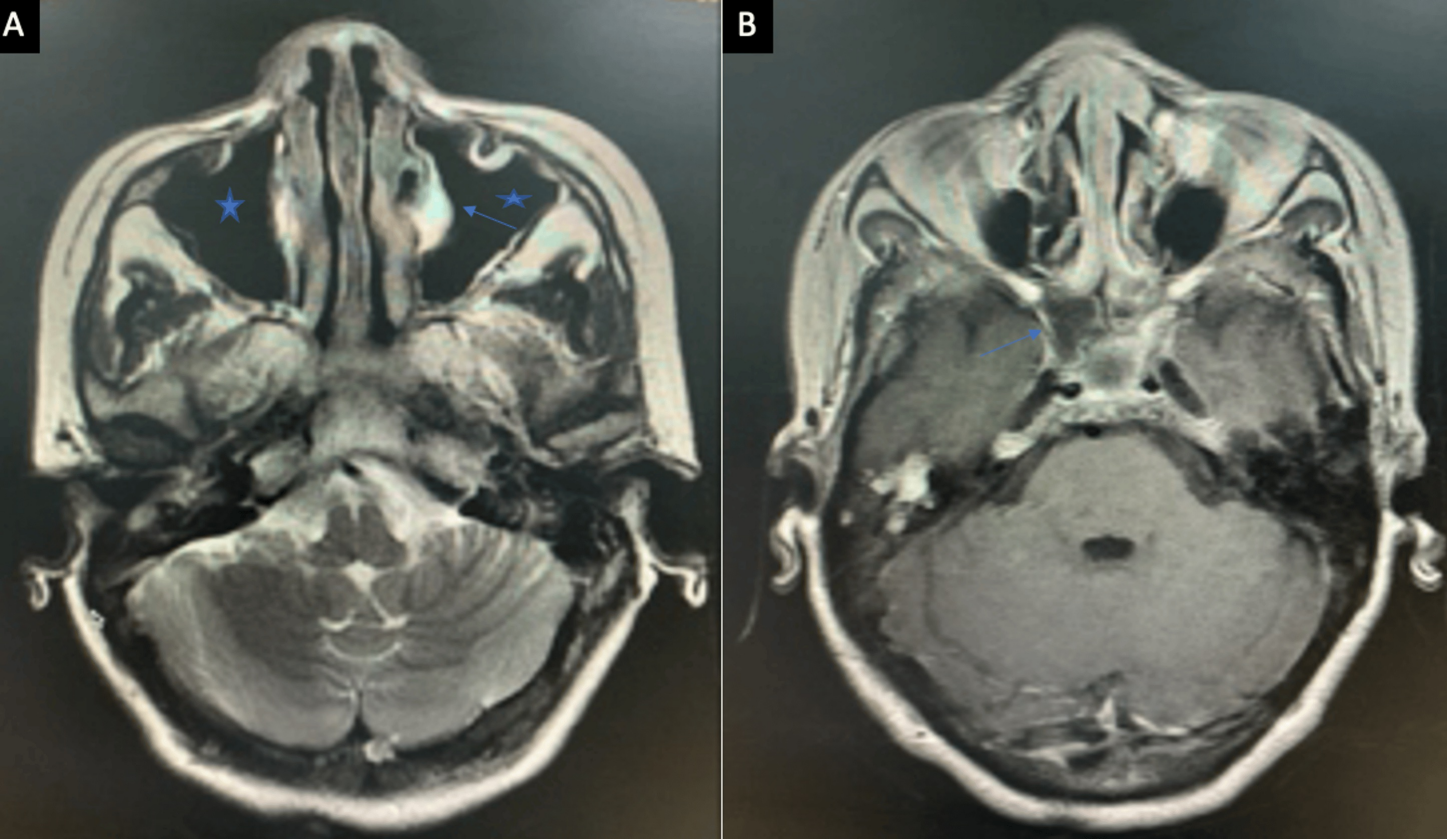
(A) A case of chronic invasive fungal infection showing mucosal thickening in bilateral maxillary sinuses (*) with mucocele in the left maxillary sinus (↗) (MRI, T2 axial). (B) Enhancing soft tissue lesion into right sphenoid sinus and extending up to the cavernous sinus (↗) (MRI, post-contrast T1 axial). MRI: magnetic resonance imaging

The maxillary sinus was the most commonly involved sinus in both acute and chronic infections. Ethmoid and sphenoid sinus involvement was also frequent (Figures 3, 4).

Nasal cavity, ethmoid sinus, frontal sinus, orbital, skull base, and cavernous sinus involvement were significantly more common in acute invasive fungal infections, underscoring the aggressive and rapidly progressive nature of the disease process (Table 2).

**Table 2:** Distribution of anatomical sites involved in imaging.

Anatomical site	Acute invasive (n = 30)	Chronic invasive (n = 30)	p-value
Nasal cavity	24 (80%)	15 (50%)	0.0149
Maxillary sinus	30 (100%)	27 (90%)	0.0756
Ethmoid sinus	29 (97%)	22 (73%)	0.0114
Sphenoid sinus	26 (87%)	20 (67%)	0.0670
Frontal sinus	22 (73%)	9 (30%)	0.0008
Orbit	26 (87%)	12 (40%)	0.0002
Skull base	24 (80%)	9 (30%)	0.0001
Cavernous sinus	14 (47%)	5 (17%)	0.0125
Intracranial involvement	16 (53%)	10 (33%)	0.1180

Imaging characteristics on CT and MRI

**Figure 5:**
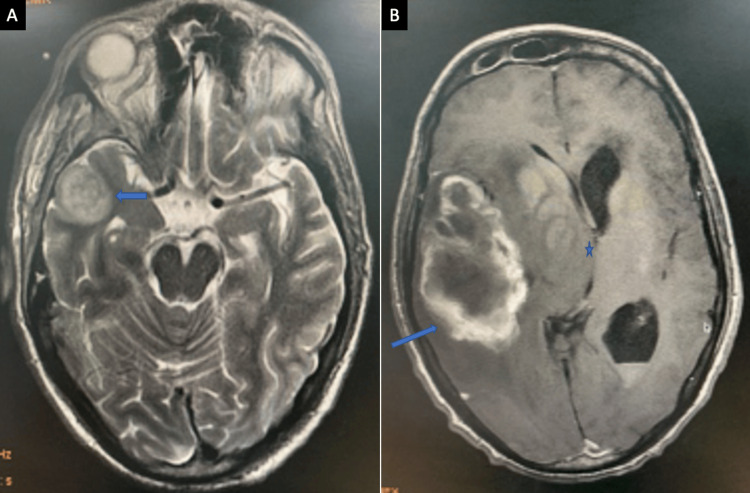
A case of acute invasive fungal infection with intracranial extension displaying a large intra-axial T2 heterogeneous signal intensity, lesion in the right temporal lobe (↗) (MRI, T2 axial). (B) Lesion is displaying irregular peripheral enhancement (↗) and significant mass effect in the form of effacement of adjacent sulci and right lateral ventricle along with midline shift toward the left side (*) (MRI, post-contrast T1 axial). MRI: magnetic resonance imaging

Hyperdense sinus contents on CT and corresponding T2 hypointense areas on MRI were significantly more common in acute invasive infections, consistent with fungal elements and necrotic tissue (Figure 5).

**Figure 6:**
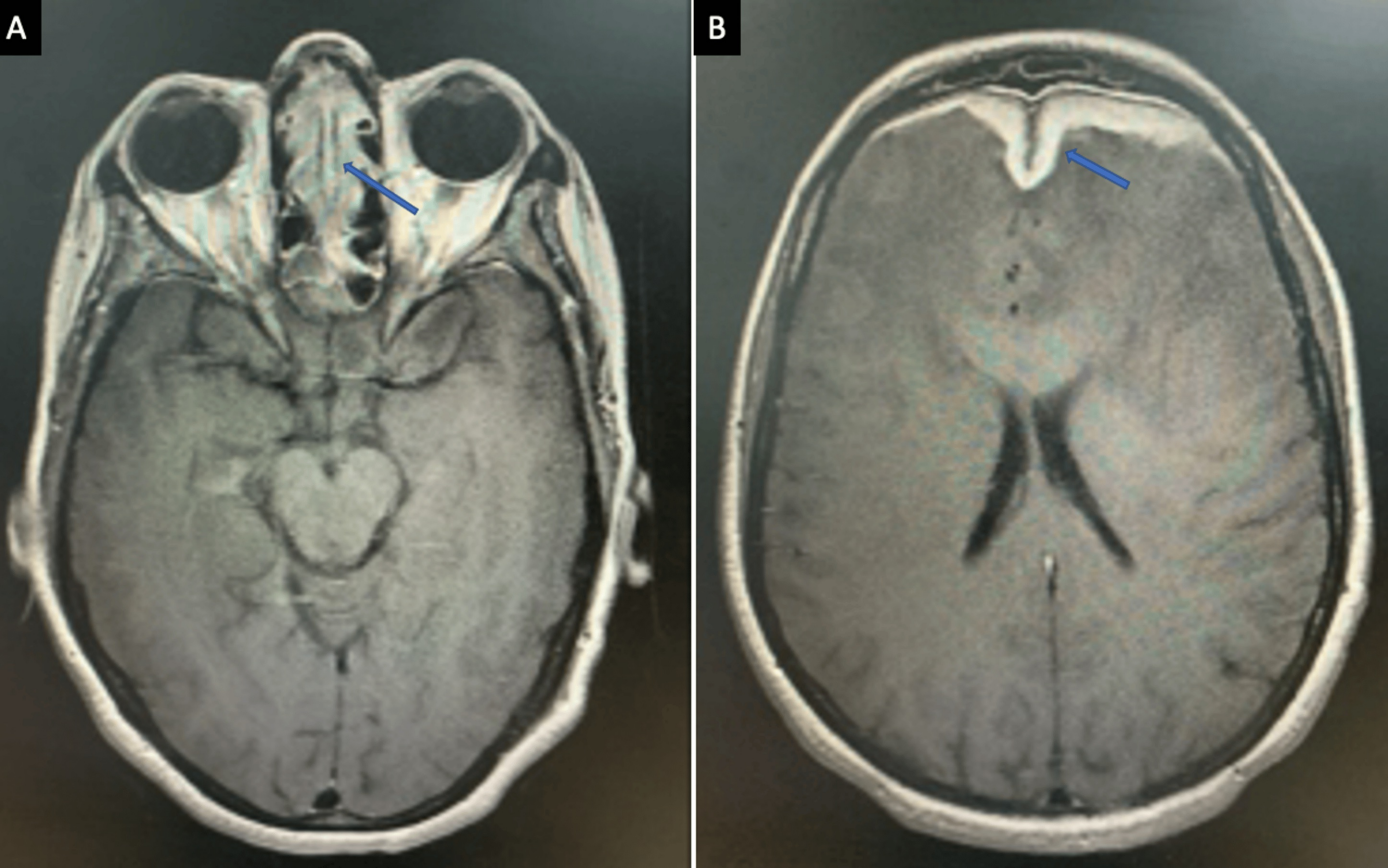
(A) A case of chronic invasive fungal infection displaying enhancing mucosal thickening in bilateral ethmoid sinuses (↗) (MRI, post-contrast T1 axial). (B) There is an intracranial extension of the disease via cribriform plate with pachymeningeal enhancement along the falx and dura of both frontal lobes (↗) (MRI, post-contrast T1 axial). MRI: magnetic resonance imaging

Extraconal and intraconal orbital involvement, optic nerve involvement, and extension into infratemporal, masticator spaces, and pterygopalatine fossa involvement were also significantly more frequent in acute disease. Chronic invasive infections more often demonstrated localized disease with less aggressive bony destruction (Figure 6; Table 3).

**Table 3:** Key computed tomography (CT) and magnetic resonance imaging (MRI) characteristics in acute and chronic invasive fungal infections.

Imaging feature	Acute invasive (n = 30)	Chronic invasive (n = 30)	p-value
Hyperdense sinus content on CT/T2 hypointensity on MRI	19 (63.3%)	8 (26.6%)	0.0043
Bone erosion	12 (40%)	6 (20%)	0.0528
Extraconal orbital involvement	24 (80%)	10 (33.3%)	<0.001
Intraconal/extraocular muscle involvement	16 (53.3%)	2 (6.6%)	0.0001
Optic nerve involvement	6 (20%)	1 (3.3%)	0.0444
Infratemporal/masticator space extension	21 (70%)	3 (10%)	0.0001
Pterygopalatine fossa involvement	8 (26.6%)	2 (6.6%)	0.0377
Dural enhancement	9 (30%)	6 (20%)	0.3711
Brain abscess/infarct	4 (13.3%)	1 (3.3%)	0.1611

Follow-up imaging response

**Figure 7:**
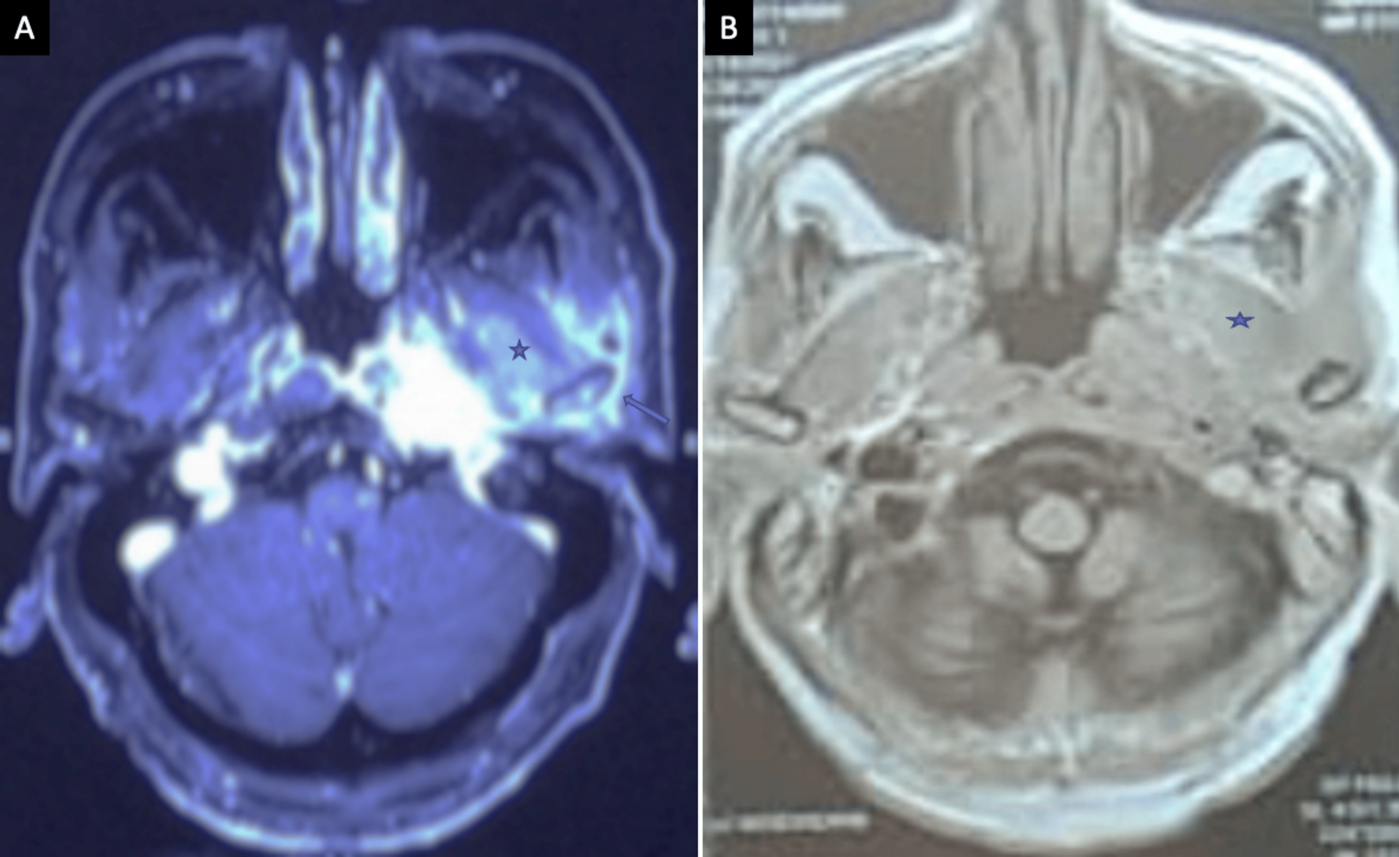
(A) A case of acute invasive skull base infection showing a heterogeneous enhancing infiltrative lesion involving the left temporomandibular joint (↗), and extension into the masticator space (*) (MRI, post-contrast T1 axial). (B) After 3 months of medical treatment, scans of the same patient showing significant interval change (*) (MRI, post-contrast T1 axial). MRI: magnetic resonance imaging

At the three-month follow-up, 76.7% (23/30) of patients with acute invasive fungal infection demonstrated radiological improvement (Figure 7).

**Figure 8:**
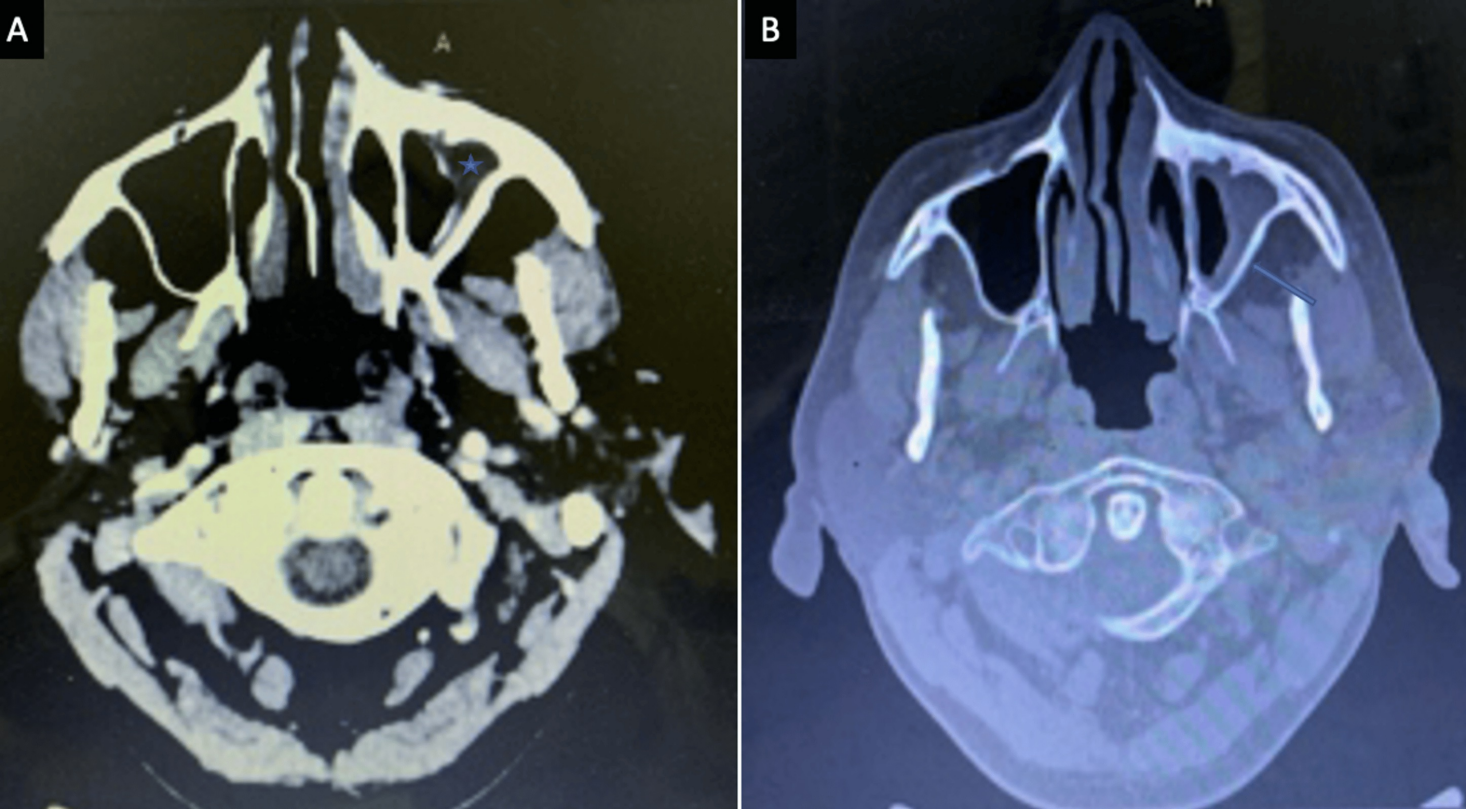
(A) A case of chronic invasive fungal infection after six months of medical treatment showing residual mucosal thickening in the left maxillary sinus (*) (CT scan, axial). (B) CT scan bone window showing sclerosis of sinus walls (↗) after six months of medical treatment (CT scan, bone window). CT: computed tomography

At the six-month follow-up, 83.3% (25/30) of chronic invasive cases showed amelioration (Figure 8).

Parameters such as reduction in lesion size or extent, normalization of signal or attenuation, and reduction in enhancement were comparable between the two groups and showed no statistically significant difference (Table 4).

**Table 4:** Follow-up imaging response after treatment.

Response parameter	Acute invasive: follow-up at 3 months (n = 30)	Chronic invasive: follow-up at 6 months (n = 30)	p-value
≥50% reduction in lesion size or extent	23 (76.7%)	25 (83.3%)	0.526
Normalization of signal/attenuation	21 (70%)	24 (80%)	0.375
>50% reduction in enhancement	20 (66.7%)	24 (80%)	0.248
Clinical improvement (≥1 symptom)	25 (83.3%)	26 (86.7%)	0.715

## Discussion

Invasive sinonasal and skull base fungal infections represent a spectrum of aggressive, life-threatening diseases whose incidence has increased substantially over the past decade, particularly in regions with a high burden of diabetes mellitus and following the COVID-19 pandemic. The present study provides a comprehensive comparison of the imaging spectrum of acute and chronic invasive fungal infections, with a particular focus on disease extent, routes of spread, complications, and radiological response to therapy. By systematically correlating CT and MRI findings with clinical and pathological data, this study reinforces the vital role of imaging not only in diagnosis but also in prognostication and planning of the treatment.

Host factors and disease biology

A key observation in this study is the striking difference in host immune status between acute and chronic invasive disease. Acute invasive fungal infections were predominantly observed in immunocompromised patients, especially those with uncontrolled diabetes mellitus and recent corticosteroid exposure. This finding is concordant with studies published in the post-COVID-19 era, which have shown a strong association between hyperglycemia and steroid-induced immune dysregulation with fungal infections [2,3,8,9]. In contrast, chronic invasive fungal infections were more commonly noted in immunocompetent patients, reflecting the indolent yet locally destructive behavior of fungal profiles. This dichotomy highlights the importance of considering host factors when interpreting imaging findings and anticipating disease behavior.

The angioinvasive nature of *Mucorales* accounts for the fulminant clinical course and the aggressive imaging appearance of acute invasive disease. Vascular invasion leads to thrombosis, ischemia, and tissue necrosis, which in turn manifest radiologically as non-enhancing devitalized mucosa, T2 hypointensity, diffusion restriction, and early extra-sinus spread [6,10]. Chronic invasive infections typically advance at a slower rate, characterized by granulomatous inflammation and fibrosis, which explains their relatively localized imaging appearance.

Sinonasal involvement and pattern of spread

In our study group, the maxillary sinus was the most common sinus affected by both acute and chronic infections. This is in line with what other studies have noted [11-13]. The maxillary sinus is a critical epicenter for disease to spread because it is very close to the nasal cavity, orbit, pterygopalatine fossa, and infratemporal fossa. Involvement of the ethmoid and sphenoid sinuses was prevalent, especially in acute invasive disease, indicating destruction of anatomical barriers.

Frontal sinus involvement was significantly more prevalent in acute invasive fungal infections. This observation is clinically significant, as frontal sinus disease frequently correlates with elevated rates of intracranial extension, including epidural abscess and frontal lobe cerebritis [14]. The existence of pansinusitis, particularly with initial bone erosion or extra-sinus soft tissue extension, should elicit a significant suspicion for acute invasive fungal infection, even in the lack of explicit clinical manifestations.

CT and MRI: complementary imaging modalities in invasive fungal rhinosinusitis

CT and MRI play different but complementary roles in the diagnosis of invasive fungal infections of the sinonasal location and the base of the skull. CT is very useful for looking at the bone structure, finding small erosions, and observing hyperdense fungal profiles in the sinuses. In our study, hyperdense sinus contents were significantly more prevalent in acute invasive disease, indicating the presence of fungal elements, hemorrhage, and necrotic debris. However, CT findings may be deceptively subtle in early disease, particularly before there is obvious bone destruction.

MRI gives better contrast for soft tissues and is necessary for screening of extra-sinus extension, vascular involvement, and intracranial complications. T2 hypointensity within involved sinuses and soft tissues, a hallmark of fungal infection due to paramagnetic elements such as iron and manganese, was significantly more frequent in acute invasive disease [5,6]. Diffusion restriction, although not systematically evaluated in this study, was frequently observed in areas of infarction, abscess formation, and devitalized tissue [15].

Orbital involvement

Orbital involvement represents a major determinant of morbidity and visual outcome in invasive sinonasal and skull base fungal infections. In the present study, orbital involvement-particularly intraconal extension, extraocular muscle infiltration, and optic nerve involvement-was significantly more common in acute invasive fungal infections. These findings are in line with those published in large post-COVID-19 series, where orbital involvement has been documented in up to 80%-90% of cases [2,10,16].

The routes of orbital spread include direct erosion of the lamina papyracea, perivascular spread through the ethmoidal vessels, and extension via the inferior orbital fissure. MRI is particularly effective at finding early signs of orbital involvement, which can happen before clinically obvious ophthalmoplegia or vision loss. Early identification of intraconal disease and optic nerve involvement is essential, as these findings frequently require immediate surgical debridement and can influence decisions pertaining to orbital exenteration.

Skull base and deep facial space involvement

Skull base involvement was a prominent feature of acute invasive disease in our study, with frequent extension into the pterygopalatine fossa, infratemporal fossa, and masticator space. These regions serve as key conduits for posterior and intracranial spread and may be involved even in the absence of extensive sinonasal disease, a phenomenon described as “occult invasive fungal infection” [7,17]. Involvement of the pterygopalatine fossa is particularly important, as it provides access to the orbit via the inferior orbital fissure and to the middle cranial fossa via the foramen rotundum and pterygoid canal. Recognition of abnormal soft tissue or loss of normal fat planes in these spaces on MRI should prompt careful evaluation for cavernous sinus and intracranial extension.

Cavernous sinus and vascular complications

Cavernous sinus and skull base involvement were observed significantly more in acute invasive fungal infections in our study cohort. Imaging findings included cavernous sinus enlargement, abnormal enhancement, loss of normal flow voids, and narrowing or thrombosis of the internal carotid artery. These findings are well-documented markers of poor prognosis and are directly related to the angioinvasive nature of mucormycosis [6,12,18].

MRI with contrast and MR angiography are necessary for identifying these complications, which may be asymptomatic in the initial stages. Early identification of vascular involvement can influence both surgical planning and the intensity of antifungal therapy.

Intracranial extension

Intracranial involvement represents the most severe end of the disease spectrum and was more common in acute invasive fungal infections in the present study. The manifestations included dural enhancement, cerebritis, brain abscess, and infarction. Chronic invasive infections exhibited intracranial extension in a subset of cases, predominantly through contiguous spread rather than vascular invasion [12].

The presence of intracranial disease significantly worsens prognosis and often necessitates prolonged antifungal therapy and multidisciplinary management with the involvement of the neurosurgery department, along with ophthalmology and the infectious disease wing. MRI remains the modality of choice for detecting early intracranial complications and monitoring treatment response [12,19].

Follow-up imaging and treatment response

The majority of patients in both groups demonstrated significant radiological improvement on follow-up, underscoring the effectiveness of combined surgical and medical management when instituted early. There was no statistically significant difference in overall radiological findings between acute and chronic infections, indicating that aggressive acute disease can be effectively managed with prompt intervention.

Follow-up imaging has several advantages, such as monitoring for residual disease, determining complications, and guiding how long to keep taking antifungal drugs. Persistent enhancement or residual soft tissue does not absolutely indicate active infection and must be assessed in conjunction with clinical manifestations and laboratory findings. Serial MRI is particularly valuable in differentiating post-treatment fibrosis from residual or recurrent disease [13,20].

The findings of this study have several important clinical implications. Primarily, radiologists must maintain a high index of suspicion for invasive fungal infection in at-risk patients presenting with sinonasal symptoms, even when imaging findings appear subtle. Second, a comprehensive imaging evaluation-including CT and contrast-enhanced MRI of the paranasal sinuses, orbit, skull base, and brain-should be performed at baseline to accurately stage disease extent.

This study has a few limitations, as the sample size is adequate for comparing results but may limit the generalizability of radiological findings. Microbiological speciation and species identification were not accessible in all cases, limiting organism-specific imaging correlations. Future multicenter studies involving larger cohorts with species identification and advanced imaging modalities may refine the precision of imaging biomarkers related to disease severity and prognosis.

## Conclusions

Invasive sinonasal and skull base fungal infections are potentially fatal conditions requiring early diagnosis and aggressive management. CT and MRI provide complementary information essential for early diagnosis and treatment of disease extent, detection of complications, and evaluation of treatment response. Acute invasive fungal infections show more aggressive imaging features and higher rates of orbital, skull base, vascular, and intracranial involvement compared to chronic invasive infections.
